# Post-COVID-19 Condition in Swiss Healthcare: Disparities in Access, Delays in Care, and Patient Burden

**DOI:** 10.3390/healthcare14142220

**Published:** 2026-07-22

**Authors:** Lara Diem, Ariane Dampfhoffer, Elisabeth Weber, Gregory Fretz, Mayssam Nehme, Iris-Katharina Penner

**Affiliations:** 1 Department of Neurology, Luzerner Kantonsspital, 6004 Lucerne, Switzerland; ariane.dampfhoffer@luks.ch; 2 Department of Internal Medicine, Stadtspital Zürich, 8063 Zurich, Switzerland; 3 Department of Internal Medicine, Cantonal Hospital of Graubünden, 7000 Chur, Switzerland; gregory.fretz@ksgr.ch; 4 Division of Primary Care Medicine, Geneva University Hospitals, 1205 Geneva, Switzerland; mayssam.nehme@hug.ch; 5 Department of Neurology, Inselspital, Bern University Hospital, University of Bern, 3012 Bern, Switzerland; iris-katharina.penner@insel.ch

**Keywords:** SARS-CoV2, coronavirus, post infectious, long-term symptoms, neuropsychiatric symptoms, viral infection

## Abstract

**Background:** Post-COVID-19 Condition (PCC) causes persistent multisystem symptoms, reduced quality of life, and high healthcare needs. Data on patient experiences in Switzerland are scarce. **Objective:** To assess healthcare access, treatment pathways, waiting times, and insurance-related burden among individuals with PCC in Switzerland. **Methods:** We conducted a nationwide anonymous online survey between July and October 2025. Adults living in Switzerland with symptoms persisting ≥12 weeks after SARS-CoV-2 infection were eligible. Recruitment was conducted through support groups, social media, and specialized PCC consultations at several Swiss hospitals. The multilingual questionnaire covered demographics, symptoms, healthcare utilization, treatment experiences, and insurance issues. **Results:** A total of 333 individuals participated (81.7% women; mean age 45.8 years). The most frequent symptoms were fatigue (98.5%), brain fog (92.5%), post-exertional malaise (91.0%), sleep disturbances (82.3%), and musculoskeletal pain (80.8%). Only 12.6% reported early and adequate medical support, whereas 45.0% had to actively seek care themselves and 24.6% had received no support. General practitioners were the most frequently consulted providers (77.5%), followed by specialized PCC clinics (39.0%). Waiting times were prolonged: 32.1% waited 4–6 months, 32.4% >6 months and 10.8% reported no available consultation service. Satisfaction with care was modest (3.02/5). Difficulties with social insurance were reported by 42.6%, including missing benefits, lack of recognition, and prolonged procedures. **Conclusions:** Individuals with PCC in Switzerland face major barriers to timely and coordinated care. Strengthening primary care competencies, expanding multidisciplinary services, and implementing structured patient-centered pathways are urgently needed to improve outcomes and reduce long-term disability.

## 1. Introduction

Post-COVID-19 Condition (PCC), commonly known as long COVID, has emerged as a major public health concern following the SARS-CoV-2 pandemic. The World Health Organization (WHO) defines PCC as symptoms that occur within three months of a probable or confirmed SARS-CoV-2 infection, last for at least two months, and cannot be explained by an alternative diagnosis [1]. PCC is characterized by a broad and heterogeneous symptom spectrum—over 200 symptoms have been described—reflecting its multisystemic nature and clinical complexity [2,3].

Estimates suggest that between 6% and 10% of all individuals infected with SARS-CoV-2 develop PCC, independent of the severity of the acute infection [4]. Among the wide range of manifestations, a few symptom clusters dominate the clinical picture. Fatigue and Post-Exertional Malaise (PEM) are reported by up to 93% of patients in specific cohorts and represent the most disabling symptoms. Other frequently reported complaints include pain syndromes (especially headache and musculoskeletal pain), sleep–wake disturbances, and neurocognitive difficulties [5]. In more severe and persistent cases, the clinical presentation often overlaps with Myalgic Encephalomyelitis/Chronic Fatigue Syndrome (ME/CFS), requiring tailored management strategies such as pacing to prevent symptom exacerbation. Prognosis in such chronic cases remains poor [6].

In Switzerland, estimation models showed that approximately 380,800 individuals were living with PCC and at least one-third of those affected are not expected to recover in the near future [7]. This burden is mirrored in social insurance data: by December 2023, around 2900 individuals with PCC had registered with the Swiss Invalidity Insurance (IV). These cases represent particularly severe disease courses, with about 90% of applicants initially classified as 100% unable to work and roughly 40% remaining fully incapacitated two years later [8].

The persistence, heterogeneity, and complexity of PCC pose substantial challenges to the Swiss healthcare and social security systems. Effective management depends on early recognition and follow-up by primary care physicians, supported by interdisciplinary and interprofessional collaboration across internal medicine and specialties such as neurology, cardiology, pulmonology, neuropsychology, occupational therapy, and physiotherapy. However, care provision remains inconsistent due to the absence of diagnostic tools for key symptoms like fatigue and effort intolerance, and fragmented access to multidisciplinary expertise. Moreover, the assessment of work capacity and disability within the IV system is often described as complex, time-consuming, and uncertain.

Despite the growing body of literature on the clinical manifestations and pathophysiology of Post-COVID-19 Condition, little is known about patients’ experiences with healthcare delivery, access to specialized services, waiting times, treatment pathways, and interactions with the social insurance system in Switzerland. To our knowledge, no nationwide study has comprehensively evaluated these patient-reported aspects of PCC care within the Swiss healthcare system. This study therefore aimed to analyze the current care situation of individuals with PCC in Switzerland through a nationwide online survey. By systematically capturing patient-reported experiences regarding diagnosis, treatment pathways, access to care, waiting times, and perceived gaps in healthcare and social insurance, this study seeks to identify key areas for improvement and inform future healthcare planning and policy development.

## 2. Methods

An online survey was conducted using the SurveyMonkey platform (https://de.surveymonkey.com/) to investigate the experiences of individuals with PCC in Switzerland. The survey was launched on 1 July 2025 and remained open until 1 October 2025. It was distributed through Swiss COVID-19 support groups (e.g., the Altea Long COVID Network), social media channels (LinkedIn and Facebook), and specialized PCC consultations at Cantonal Hospital of Lucerne, University Hospital Bern (Inselspital), Geneva University Hospitals, Stadtspital Zürich Waid, and Cantonal Hospital of Graubünden.

Prior to participation, all respondents provided digital informed consent to this anonymous survey. The Ethics Committee of Northwestern and Central Switzerland (EKNZ) was informed about the project and determined that it did not require formal ethical approval, as it did not fall within the scope of the Swiss Human Research Act (Art. 2, Para. 1; Req-2024-00517).

The questionnaire was originally designed in German and subsequently translated into French and Italian by native bilingual speakers. Conditions for participation were clearly stated in all three languages: participants were eligible if (1) their acute COVID-19 illness had occurred at least 12 weeks prior to participation and (2) they were 18 years or older.

The questionnaire was specifically developed for this study, as no validated instrument was available to comprehensively assess healthcare utilization, treatment pathways, waiting times, and social insurance experiences among individuals with PCC in Switzerland. Its development was informed by the current literature, Swiss PCC recommendations, and the clinical experience of an interdisciplinary team. Prior to distribution, the questionnaire was pilot-tested for clarity, comprehensibility, and face validity, resulting in minor wording adjustments. As the survey consisted mainly of independent descriptive items and a limited number of open-ended questions, formal assessment of internal consistency was not applicable.

The survey comprised 17 items covering sociodemographic characteristics (age, sex, canton of residence), infection history (number and year of COVID-19 infections, severity), symptom profiles, course of illness, and healthcare experiences. Specific questions addressed access to medical care, waiting times for specialized consultation, types of therapies received (e.g., physiotherapy, occupational therapy, psychotherapy, pharmacologic and off-label treatments), and interactions with insurance systems (e.g., IV, SUVA). Participants could also provide open-text responses regarding perceived care gaps and suggestions for improvement.

Data are presented as mean with 95% confidence intervals (95% CI); comparative statistics were applied, and the type of testing is mentioned prior to each *p*-value. Continuous variables were compared using Mann–Whitney U test, as appropriate, and categorical variables using Pearson’s χ^2^ test. Given the exploratory nature of these analyses, no adjustment for multiple testing was applied. A *p*-value of 0.05 was considered significant.

Statistical analyses were performed using IBM SPSS Statistics version 30 (IBM Corp., Armonk, NY, USA).

Data sharing statement: Following an open data approach, anonymized data of the cohort can be requested via the corresponding author.

Statement on funding sources and conflicts of interest: This research received no specific grant from any funding agency in the public, commercial, or not-for-profit sectors. The authors declare that there are no conflicts of interest regarding the publication of this paper.

## 3. Results

A total of 333 individuals with PCC participated in the survey. The cohort was strongly female-dominated, with 272 of 333 participants (81.7%) identifying as women, and had a mean age of 45.8 years (95% CI 44.6–47.3). Geographically, the largest proportion of participants resided in the canton of Zurich (85/333, 25.5%), followed by Bern (50/333, 15.0%) and Lucerne (33/333, 9.9%).

On average, participants reported 2.2 SARS-CoV-2 infections (95% CI 2.1–2.3). The first infection occurred most frequently in 2022 (143/333, 42.9%), followed by 2020 (89/333, 26.7%) and 2021 (76/333, 22.8%). Most respondents described their acute COVID-19 phase as moderate (259/333, 77.8%), while severe cases requiring hospitalization (17/333, 5.1%) or ICU admission (3/333, 0.9%) were rare.

The burden of PCC symptoms was substantial. Nearly all participants reported fatigue (319/333, 98.5%). Difficulty concentrating/brain fog was present in 308 participants (92.5%), and post-exertional malaise (PEM) in 303 (91.0%). Other frequent symptoms included sleep disorders (274/333, 82.3%), photo- or phonophobia (272/333, 81.7%), joint and muscle pain (269/333, 80.8%), and headache (239/333, 71.8%). Less frequent were palpitations/blood-pressure fluctuations (233/333, 70.0%), dyspnea (187/333, 56.2%), and gastrointestinal symptoms (195/333, 58.6%). Formal diagnoses were relatively rare within the cohort, with postural tachycardia syndrome (POTS) identified in 18 of 333 patients (5.4%) and small-fiber polyneuropathy in 7 of 333 patients (2.1%); however, symptoms suggestive of autonomic dysfunction were reported far more frequently.

Regarding the course of illness, 133 participants (39.9%) reported symptomatic improvement, 103 (30.9%) complete remission, 31 (9.3%) unchanged symptoms, and 66 (19.8%) a worsening of their condition.

Access to medical care was limited. Only 42 participants (12.6%) stated that they received early and good support, whereas 150 (45.0%) independently sought specialist care, and 82 (24.6%) had still received none. The general practitioner was by far the most frequently consulted provider (258/333, 77.5%), followed by specialists such as neurologists or pulmonologists (106/333, 31.8%) and specialized PCC consultation hours (130/333, 39.0%).

Waiting times for specialized consultation were long: 107 participants (32.1%) waited 4–6 months, 108 (32.4%) waited more than 6 months, and 36 (10.8%) reported that no such consultation was available. The mean satisfaction score with medical care was 3.02 (95% CI 2.9–3.2) on a 1–5 scale.

Regarding treatment, the majority received physiotherapy (249/333, 74.8%) and ergotherapy (220/333, 66.1%). Psychotherapy was used by 191 (57.4%), relaxation or complementary medicine by 164 (49.2%), and rehabilitation by 114 (34.2%). Symptomatic medication was prescribed in 193 cases (58.2%), and off-label pharmacological treatments such as low-dose naltrexone or antihistamines were reported by an identical proportion (193/333, 58.0%). Invasive off-label treatments like apheresis were rare (23/333, 6.9%). Only 127 participants (38.1%) received specific treatment recommendations from their physician, while 160 (48.0%) reported that they obtained such advice only by using their own initiative.

Overall, 142 of 333 respondents (42.6%) reported difficulties with social insurance, most commonly related to missing benefits from health insurance (13.5%) as well as lack of recognition of the diagnosis and prolonged administrative processes (both 10.5%); in addition, 7.2% reported a deterioration in their condition following expert assessments, and 0.9% experienced non-payment by daily allowance insurance. Detailed baseline characteristics are presented in Table 1.

Analysis of the 283 open-text responses identified several recurring themes regarding improvements in PCC care (Supplementary Table S1). The most frequently reported suggestions included better education of healthcare professionals, expansion of specialized multidisciplinary PCC clinics, improved social insurance processes, greater recognition of PCC by healthcare providers and insurers, increased research efforts, improved access to evidence-based and off-label treatments, and better support for severely affected or housebound patients through home-based or telemedicine services.

Exploratory subgroup analyses comparing participants with and without complete symptom remission showed that remission was significantly associated with earlier access to medical care (47.0% vs. 19.1%, *p* < 0.001). In contrast, participants without remission more frequently reported insurance-related difficulties (39.7% vs. 54.5%, *p* = 0.029) and were more likely to have received specific treatment recommendations (40.8% vs. 27.3%, *p* = 0.042). No significant differences were observed regarding age, sex, number or severity of SARS-CoV-2 infections, attendance at specialized PCC consultations, or the use of off-label therapies (Table 2).

Participants who reported being satisfied with their medical care more frequently received specific treatment recommendations (53.0% vs. 28.4%, *p* < 0.001) and off-label therapies (65.9% vs. 53.7%, *p* = 0.027). No significant differences were observed regarding early access to care, attendance at a specialized PCC consultation, or insurance-related difficulties (Table 3).

## 4. Discussion

This nationwide survey provides the first comprehensive patient-reported overview of PCC care in Switzerland, revealing substantial shortcomings in access, coordination, and timeliness of medical support. The findings demonstrate that a majority of affected individuals experience significant barriers in obtaining adequate care, reflecting both the systemic unpreparedness of the healthcare system and the persistent uncertainty surrounding this complex condition.

### 4.1. Demographic and Clinical Characteristics

The demographic and clinical characteristics of our cohort are largely consistent with the current epidemiological evidence on PCC [2]. The marked female predominance reflects the known epidemiology of PCC, as women are more frequently affected than men [9]. Furthermore, most participants experienced only a mild to moderate acute SARS-CoV-2 infection, supporting previous observations that persistent symptoms frequently develop even after non-severe acute disease. The symptom profile closely mirrors that reported in international cohorts, with fatigue, cognitive dysfunction (brain fog), and post-exertional malaise representing the predominant clinical manifestations, followed by sleep disturbances, pain syndromes, headache, and symptoms suggestive of autonomic dysfunction [5]. Interestingly, while formal diagnoses such as POTS and small-fiber polyneuropathy were relatively uncommon, autonomic symptoms were reported considerably more frequently, suggesting that autonomic dysfunction may remain underrecognized or underdiagnosed in routine clinical practice. Overall, the close agreement between our findings and previous international studies supports the external validity of our cohort and provides a robust basis for interpreting the reported healthcare experiences.

### 4.2. Deficits in Access and Continuity of Care

Only 12.6% of respondents reported early and good medical support, while nearly half (45.0%) had to actively seek care, and one quarter (24.6%) had not received any medical support at all.

Exploratory subgroup analyses further demonstrated that participants who achieved complete symptom remission were significantly more likely to have received early medical care than those with persistent symptoms. This finding supports the concept that timely recognition and early management may contribute to more favorable clinical outcomes, although causal relationships cannot be inferred from this cross-sectional study.

Overall satisfaction with the care and treatment was rated as low, at 3.02. This lack of structured access led many patients to independently navigate their treatment pathways, as reflected by the 48.0% who reported receiving therapeutic recommendations only on their own initiative. Waiting times for specialized consultations were unacceptably long: over 64% waited over four months, and specialized PCC clinics were unavailable for approximately 10% of participants.

These findings suggest insufficient capacity and fragmentation of PCC care structures, particularly the limited number of multidisciplinary outpatient clinics and rehabilitation programs. The observed lack of early medical support, prolonged waiting times, and limited availability of specialized services suggest that the current availability of specialized PCC services may not fully meet patients’ needs. Specialized PCC clinics play an important role in providing multidisciplinary assessment, diagnostic clarification, and coordinated management for complex cases that cannot always be adequately addressed in primary care alone. The resulting care gap likely contributes to chronicity and worsened functional outcomes, as timely recognition and early rehabilitation are known determinants of prognosis in PCC [5]. Maintaining and further developing specialized services—ideally embedded within structured regional care networks and closely linked to primary care—may help improve timely access to care and reduce existing gaps in the management of individuals with PCC, post-infectious conditions, and chronic fatigue syndromes.

### 4.3. Complexity and Multisystem Involvement

The heterogeneous and multisystem nature of PCC—encompassing fatigue, cognitive dysfunction, exertional intolerance, pain, and autonomic symptoms—poses unique diagnostic and therapeutic challenges. The absence of specific biomarkers and validated diagnostic algorithms complicates clinical differentiation from other chronic conditions such as depression, fibromyalgia, or ME/CFS [10]. This diagnostic uncertainty often leads to inconsistent recognition across specialties and frustration among both patients and providers. The situation is aggravated by the lack of standardized management pathways and limited awareness among healthcare professionals, despite increasing evidence for the importance of structured pacing, graded rehabilitation, and psychological support [11]. In addition, diagnostic ambiguity may lead some patients to seek repeated opinions across disciplines, which can erode continuity of care and further increase strain on both sides; through disrupted follow-up, repeated investigations, and heightened stress, this may contribute to symptom persistence or even exacerbation.

### 4.4. General Practitioners as Gatekeepers in Post-COVID-19 Care

General practitioners (GPs) are central to the management of PCC care, given the high prevalence of persistent symptoms and the multisystem nature of the condition [5,9,12]. In our study, GPs were by far the most frequently consulted healthcare providers, with 258 of 333 respondents (77.5%) reporting contact with their GP, compared with 31.8% who consulted medical specialists such as neurologists or pulmonologists and 39.0% who accessed specialized PCC consultation services. This finding underscores the role of GPs as the primary first point of contact and gatekeepers in PCC care pathways, highlighting that early recognition, initial management, and longitudinal support largely depend on primary care structures [5,13].

Key elements of GP-led care include a comprehensive clinical history covering premorbid functioning, details of the acute SARS-CoV-2 infection, and symptom evolution, complemented by careful clinical examination and validation of the patient’s experience [14]. The use of condition-specific patient-reported outcome measures (PROMs) is recommended to monitor disease burden and symptom trajectories over time [5,14]. In addition, GPs are responsible for identifying red flag conditions, managing comorbidities, providing education and reassurance, and initiating symptom-targeted interventions. A structured, stepwise approach within primary care—based on regular follow-up, functional assessment, and early supportive measures—is essential to prevent diagnostic delay and therapeutic inertia [13].

Non-pharmacological strategies, including pacing and energy management for fatigue, physiotherapy for respiratory and functional limitations, psychological support, cognitive rehabilitation, and olfactory training, form the cornerstone of management, while pharmacological treatments should be reserved for clearly defined symptoms and follow established disease-specific guidelines within an individualized and predominantly conservative treatment framework [11,15,16].

Despite this pivotal role, GPs face substantial challenges in delivering PCC care. Two main barriers can be identified. First, the heterogeneity and fluctuating multisystem presentation of symptoms, combined with the absence of specific biomarkers or validated diagnostic algorithms, creates significant diagnostic uncertainty and complicates risk stratification and prognostication, making clinical decision-making particularly demanding in time-limited primary care consultations [5,13]. Second, many GPs lack sufficient structural and time resources to manage complex and often prolonged PCC cases within routine primary care, a limitation that is further aggravated by workforce shortages, increasing part-time work, and high administrative burden [15,17]. This situation is further exacerbated by the growing shortage of general practitioners in Switzerland. More than three quarters of practicing GPs currently report regional shortages in primary care provision, with rates exceeding 90% in peripheral regions [18].

As a consequence, patients are frequently referred early to specialized PCC clinics, often without substantial interim management being initiated in primary care. In addition, participants who reported higher satisfaction with their medical care were significantly more likely to have received specific treatment recommendations and off-label therapies. This finding suggests that active therapeutic management and individualized treatment strategies may positively influence patients’ perception of care, even in the absence of established disease-modifying therapies. While appropriate in selected cases, this referral-driven approach may lead to delays in active care, fragmented patient pathways, and an increased risk of symptom persistence and chronification, given that early recognition and timely supportive interventions are key determinants of prognosis in PCC [5,16].

Beyond the well-recognized shortage of general practitioners, enhancing primary care requires strengthening disease-specific knowledge and competencies and establishing a streamlined structured initial assessment tool which GPs can easily implement.

Our findings suggest that strengthening disease-specific knowledge and providing practical management tools for general practitioners may facilitate earlier recognition, improve continuity of care, and reduce unnecessary delays in referral to specialized services.

### 4.5. Impact on Social Security and Work Capacity

The consequences of these care deficits extend into the social insurance system. Applicants to the Swiss Invalidity Insurance (IV) typically represent the most severely affected group, with pronounced fatigue and neurocognitive dysfunction. At the time of registration, 90% were classified as fully unable to work, and many remained so after two years. The subjective and fluctuating nature of key symptoms, particularly fatigue and effort intolerance, renders the assessment of work capacity complex and prone to inconsistency. These findings emphasize the need for medically informed evaluation frameworks within the IV system and for close interdisciplinary and interprofessional collaboration between clinicians, insurers, and rehabilitation specialists to ensure fair and efficient case assessment.

Although our survey was not designed to evaluate specific interventions within the social insurance system, the high proportion of participants reporting insurance-related difficulties suggests several potential areas for improvement. First, standardized, medically informed assessment frameworks for PCC within the Swiss Invalidity Insurance (IV) system may facilitate a more consistent evaluation of fluctuating symptoms, post-exertional symptom exacerbation, and cognitive fatigability, which are often difficult to capture in single-time-point assessments. Second, closer interdisciplinary and interprofessional collaboration between treating physicians, occupational health specialists, rehabilitation professionals, psychologists, and IV case managers may improve consistency and transparency in work-capacity assessments. Third, the use of validated patient-reported outcome measures and functional assessment tools, particularly those assessing fatigue severity, cognitive load, and exertional intolerance, may support more comprehensive longitudinal evaluations. Finally, early vocational rehabilitation and symptom-adapted work reintegration, aligned with pacing principles where appropriate, may help facilitate sustainable work participation in individuals with PCC.

### 4.6. Need for Coordinated Multidisciplinary Care

Given the multisystemic presentation of PCC, a multidisciplinary model integrating general practitioners, internal medicine, neurologists, psychologists, pulmonologists, rehabilitation specialists, and mental health professionals is essential [19]. Coordination remains a major challenge in the Swiss context, where healthcare organization is highly decentralized and specialized PCC clinics are scarce. Expanding therapeutic capacity, particularly in physiotherapy, occupational therapy, and cognitive rehabilitation, is crucial to prevent secondary deconditioning and social isolation. Targeted education initiatives, such as those initiated by the Swiss Neurological Society (SNG) Task Force, can help address uncertainty among practitioners and foster a shared clinical understanding of PCC.

### 4.7. Towards Patient-Centered Implementation

Beyond medical infrastructure, the survey underscores a fundamental need for patient-centered care that validates symptoms, ensures continuity, and integrates psychosocial support. Empathy, active listening, and consistent follow-up remain therapeutic tools in their own right, especially in conditions where pathophysiology and prognosis are still incompletely understood [12]. The forthcoming national long COVID strategy mandated by the Swiss Parliament in 2025 offers a critical opportunity to anchor these principles in policy and practice—but meaningful change will depend on their implementation at the clinical front line.

### 4.8. Study Limitations

This study has some limitations that should be considered when interpreting the findings. First, its cross-sectional design precludes conclusions regarding causal relationships between healthcare structures, treatment pathways, and patient outcomes. Second, all data were self-reported and were not independently verified by medical records, which may have introduced reporting and recall bias. Furthermore, symptom severity, diagnoses, and healthcare experiences were based on participants’ own perceptions.

Third, recruitment through patient support groups, social media platforms (Facebook and LinkedIn), and specialized Post-COVID consultation clinics may have introduced selection bias. Individuals with more severe or persistent symptoms, those actively seeking healthcare, patients dissatisfied with previous medical care, and participants with higher digital literacy were likely overrepresented, whereas individuals with milder disease or complete recovery may have been less likely to participate. Consequently, the study population may not fully represent the entire spectrum of individuals living with Post-COVID-19 Condition in Switzerland, thereby limiting the generalizability of our findings.

In addition, the marked predominance of female participants may have influenced the observed symptom distribution and healthcare experiences. However, this sex distribution is consistent with previous epidemiological studies demonstrating that women are more frequently affected by Post-COVID-19 Condition than men.

Finally, the open recruitment strategy precluded calculation of a response rate, limiting the assessment of the sample representativeness. Nevertheless, despite these limitations, this study provides one of the first nationwide patient-reported overviews of healthcare access, treatment pathways, waiting times, and social insurance experiences among individuals with Post-COVID-19 Condition in Switzerland.

## 5. Conclusions

This nationwide survey reveals substantial and persistent gaps in PCC care in Switzerland, characterized by delayed access, fragmented pathways, and insufficient coordination between primary, specialized, and social insurance systems. Our findings suggest that strengthening GP-led care, improving coordination between healthcare providers, and ensuring adequate access to multidisciplinary services may help reduce delays in care and improve patient outcomes.

## Figures and Tables

**Table 1:** Demographic Characteristics, Clinical Features, Healthcare Utilization, and Social Insurance Aspects of Participants with Post-COVID-19 Condition (*n* = 333).

Variable	
Age, Mean (95% CI), *n*	45.8 (44.6–47.3), 333
Female sex, *n* (%)	272/333 (81.7)
Canton, *n* (%)	
Aargau	22/333 (6.6)
Appenzell Innerrhoden	0/333
Appenzell Ausserrhoden	5/333 (1.5)
Bern	50/333 (15.0)
Basel-Landschaft	16/333 (4.8)
Basel-Stadt	9/333 (2.7)
Fribourg	5/333 (1.5)
Geneva	3/333 (0.9)
Glarus	4/333 (1.2)
Grisons	18/333 (5.4)
Jura	0/333
Lucerne	33/333 (9.9)
Neuchâtel	2/333 (0.6)
Nidwalden	2/333 (0.6)
Obwalden	4/333 (1.2)
St. Gallen	29/333 (8.7)
Schaffhausen	3/333 (0.9)
Solothurn	12/333 (3.6)
Schwyz	4/333 (1.2)
Thurgau	11/333 (3.3)
Ticino	0/333
Uri	2/333 (0.6)
Vaud	4/333 (1.2)
Valais	5/333 (1.5)
Zug	5/333 (1.5)
Zürich	85/333 (25.5)
Date of first SARS-CoV-2 infection, *n* (%)	
2020	89/333 (26.7)
2021	76/333 (22.8)
2022	143/333 (42.9)
2023	20/333 (6.0)
2024	4/333 (1.2)
2025	1/333 (0.3)
Number of infections, mean (95% CI), *n*	2.2 (2.1–2.3), 333
Severity of acute SARS-CoV-2 infection, *n* (%)	
asymptomatic	2/333 (0.6)
mild	52/333 (15.6)
moderate	259/333 (77.8)
severe with hospitalization	17/333 (5.1)
ICU	3/333 (0.9)
Symptoms of Post-COVID-19 Condition (PCC), *n* (%)	
Dyspnea	187/333 (56.2)
Fatigue	319/333 (98.5)
Difficulty concentrating/brain fog	308/333 (92.5)
Joint and muscle pain	269/333 (80.8)
Headache	239/333 (71.8)
Palpitations, fluctuations in blood pressure	233/333 (70.0)
Diagnosis of Postural tachycardia syndrome (POTS)	18/333 (5.4)
Post-exertional malaise	303/333 (91.0)
Photo-/Phonophobia	272/333 (81.7)
Sleep disorders	274/333 (82.3)
Gastrointestinal symptoms	195/333 (58.6)
Smell and taste disorders	124/333 (37.2)
Tinnitus	152/333 (42.9)
Neuropathic pain	143/333 (42.9)
Diagnosis of small-fiber polyneuropathy	7/333 (2.1)
Subfebrile temperature/lymph node swelling	94/333 (28.2)
Dermatological symptoms	11/333 (3.3)
Visual disturbance	3/333 (0.9)
Gynecological complaints	4/333 (1.2)
Course of symptoms, *n* (%)	
Unchanged	31/333 (9.3)
Better	133/333 (39.9)
Remission	103/333 (30.9)
Worsening	66/333 (19.8)
Medical support, *n* (%)	
Still none	82/333 (24.6)
Early and good	42/333 (12.6)
Had to be actively sought	150/333 (45.0)
Long awaited	59/333 (17.7)
Type of healthcare provider, *n* (%)	
General practitioner	258/333 (77.5)
Specialist (neurologist, pneumologist, etc.)	106/333 (31.8)
Rehabilitation clinic	57/333 (17.1)
PCC consultation hour	130/333 (39.0)
none	27/333 (8.1)
How well do you feel cared for by your doctor? (1–5), mean (95%CI) *n*	3.02 (2.9–3.2), 333
Waiting time for specialized consultation, *n* (%)	
No consultation hours available	36/333 (10.8)
<1 month	18/333 (5.4)
1–3 months	64/333 (19.2)
4–6 months	107/333 (32.1)
>6 months	108/333 (32.4)
Type of therapy received *n* (%)	
None	13/333 (3.9)
Physiotherapy	249/333 (74.8)
Ergotherapy	220/333 (66.1)
Neuropsychology/cognitive training	44/333 (13.2)
Psychotherapy	191/333 (57.4)
Rehabilitation	114/333 (34.2)
Respiratory therapy	51/333 (15.3)
Relaxation technique/complementary medicine	164/333 (49.2)
Medication (symptomatic treatment)	193/333 (58.2)
Off-label medication (LDN, antihistamines…)	193/333 (58.0)
IVIG	3/333 (0.9)
Use of off-label invasive therapies (HELP apheresis, immunoabsorption, plasmapheresis)	23 (6.9)
Specific treatment recommendations, *n* (%)	
Yes	127/333 (38.1)
No	46/333 (13.8)
Only on one’s own initiative	160/333 (48.0)
Difficulties with social insurance, *n* (%)	142/333 (42.6)
Type of social insurance difficulty, *n* (%)	
No recognition of the diagnosis	35/333 (10.5)
Long process	35/333 (10.5)
Deterioration due to expert consultation	24/333 (7.2)
No payment by daily allowance insurance	3/333 (0.9)
Missing benefits from health insurance	45/333 (13.5)

Data are presented as mean (95% confidence interval, CI) or number (percentage, %). *n* indicates the number of participants. PCC refers to persistent or newly emerging symptoms lasting ≥3 months after acute SARS-CoV-2 infection without an alternative explanation. ICU = intensive care unit; POTS = postural tachycardia syndrome; IVIG = intravenous immunoglobulins; LDN = low-dose naltrexone; HELP apheresis = heparin-induced extracorporeal LDL precipitation.

**Table 2:** Comparison of participants with and without symptom remission.

Variable	No Remission	Remission	*p*-Value
Clinical Characteristics			
Age	46.1 (44.5–47.6), 267	45.4 (42.1–48.6), 66	0.863
Female Sex	222/267	50/66	0.165
Number of infections	2.2 (2.1–2.3), 267	2.1 (1.9–2.3), 66	0.334
Severe infection	18/267	2/66	0.256
Management			
Early care (≤3 months)	51/267	31/66	**<0.001**
Satisfaction with the care provided (1–5)	3.1 (2.9–3.3), 267	2.7 (2.4–3.1),66	**0.049**
Attended a specialist consultation	102/267	28/66	0.529
Problems with insurance	106/267	36/66	**0.029**
Treatment			
Specific treatment	109/267	18/66	**0.042**
Off-label therapies	154/267	41/66	0.512

Data are presented as mean (95% confidence interval) or number (%). Remission was defined as complete resolution of Post-COVID-19 Condition symptoms at the time of the survey. Comparisons between groups were performed using the independent-samples Mann–Whitney U test for continuous variables and Pearson’s χ^2^ test for categorical variables, as appropriate. *p* values < 0.05 were considered statistically significant. Statistically significant *p*-values are shown in bold.

**Table 3:** Factors associated with patient satisfaction with medical care.

Variable	Not Satisfied	Satisfied	*p*-Value
Early care (≤3 months)	43/201	39/132	0.091
Attended a specialist consultation	79/201	51/132	0.903
Problems with insurance	87/201	55/132	0.770
Specific treatment	57/201	70/132	**<0.001**
Off-label therapies	108/201	87/132	**0.027**

Data are presented as number (%) of participants. Satisfaction with medical care was dichotomized into not satisfied (scores 1–3) and satisfied (scores 4–5) based on the 5-point Likert scale. Comparisons between groups were performed using Pearson’s χ^2^ test, as appropriate. *p* values are reported descriptively and should be interpreted as exploratory; no adjustment for multiple comparisons was applied. Statistically significant *p*-values are shown in bold.

## Data Availability

The anonymized data supporting the findings of this study are available from the corresponding author upon reasonable request. The data are not publicly available due to privacy and ethical considerations.

## Supplementary Materials

The following supporting information can be downloaded at: https://www.mdpi.com/article/10.3390/healthcare14142220/s1, Table S1: Themes identified from the open-text responses regarding suggested improvements in Post-COVID-19 Condition care in Switzerland, including illustrative patient quotations.

## Abbreviations

The following abbreviations are used in this manuscript:

CI	Confidence Interval
COVID-19	Coronavirus Disease 2019
EKNZ	Ethics Committee of Northwestern and Central Switzerland
GP	General Practitioner
ICU	Intensive Care Unit
IV	Swiss Invalidity Insurance
ME/CFS	Myalgic Encephalomyelitis/Chronic Fatigue Syndrome
PCC	Post-COVID-19 Condition
PEM	Post-Exertional Malaise
POTS	Postural Orthostatic Tachycardia Syndrome
PROMs	Patient-Reported Outcome Measures
SARS-CoV-2	Severe Acute Respiratory Syndrome Coronavirus 2
SUVA	Swiss National Accident Insurance Fund
WHO	World Health Organization
