# Supplementary material for: Post-COVID-19 Condition in Swiss Healthcare: Disparities in Access, Delays in Care, and Patient Burden
Source: Healthcare (Basel). 2026 Jul 22;14(14):2220. doi: 10.3390/healthcare14142220 (PMC13409836; doi:10.3390/healthcare14142220)
Supplement: Supplementary file 1 [file healthcare-14-02220-s001.zip › healthcare-4374207-supplementary.pdf]

Supplementary Table S1.
Main patient-reported suggestions for improving PCC care in Switzerland based on open-text responses (n = 283)

Theme identified in open-text responses	Description / examples	n	% of responses
Education and training of healthcare professionals	Better training of GPs, specialists, therapists and nursing staff regarding PCC, ME/CFS, PEM, POTS and pacing	147	51.9%
Specialized PCC clinics / competence centers	More specialized outpatient clinics, competence centers, long-term follow-up and multidisciplinary care	131	46.3%
Social insurance and financial support	Faster and more humane IV procedures, better insurance recognition, financial support, adapted assessments	80	28.3%
Recognition and validation of PCC	Being taken seriously, less psychologization, reduced stigma, recognition as a somatic condition	77	27.2%
More research and drug development	More clinical studies, research funding, medication trials and registry data	62	21.9%
Access to treatment and reimbursement	Coverage of therapies, off-label medication, complementary treatments and individualized treatment options	39	13.8%
Home-based / telemedicine care	Home visits, online consultations, support for housebound or severely affected patients	36	12.7%
PEM/pacing and avoidance of harmful activation	Better awareness of PEM, pacing, avoidance of inappropriate activation or unsuitable rehabilitation	34	12.0%
Public awareness and information	Better information for the public, schools, employers, authorities and media	22	7.8%
Pediatric and adolescent care	Better care pathways for children, adolescents, schools and families	20	7.1%
Coordination / case management	Central coordination, case management, interdisciplinary networks and improved information flow	19	6.7%
